# Cure of Hydrocephalus by Means of Hydriodate of Potassa

**Published:** 1844-03

**Authors:** 


*Cure of Hydrocephalus by means of large doses of Hydriodate of Potassa.* By Dr. Woeniger.—A child nearly two years old, of a scrofulous and lymphatic constitution, and who had before suffered from affections of the head and eyes, was attacked in August, 1841, after some days indisposition, with a severe inflammation of the brain. Dr. W. first saw him on the 25th August, when he commenced a severe antiphlogistic treatment; eight leeches were ordered to the back of the neck, and the bleeding from the bites was long maintained; followed by a large blister to the neck, upon which free suppuration was kept up, ice-water in a bladder to the shaved head, mustard plasters to the calf of the legs, and powders of 1 gr. of calomel with jalap, every two hours. Besides this, neapolitan ointment was freely rubbed upon the top of the head and neck.

"No improvement was observed, notwithstanding the continued employment of the above means, and repeated applications of leeches, although several passages from the bowels were obtained. On the contrary, the child grew worse, and on the 5th day, unmistakeable signs of water in the ventricles manifested themselves: namely, fixed and watery eyes, immovable and widely-dilated pupil, entire blindness, tetanic rigidity of the muscles of the neck, retraction of the head, paralysis of the left extremities, deep stupor, pulse slow (50 beats), having before been very small and quick, frequent screeching and vomiting, &c. In this desperate condition, Dr. W. recollecting the recommendations of large doses of hyd. pot. in such cases by Dr. Röser, laid aside all other means and prescribed R. hydriod. pot. ʒj., aquæ distillat. ℥ss. of which 40 drops were to be taken every two hours, in sugared water; and on the next day as much as 50 drops each dose. During the first three days no improvement was manifested, but upon the fourth, (the ninth of the disease), there was a decided remission of several of the unpleasant symptoms, and especially a free secretion of urine, which had been very scanty before From this time, and after nearly ʒij. of hyd. pot. had been taken, a rapid improvement was observed, and by the 12th day, the child was considered as cured. It must also be remarked that, at the beginning of the attack, there was observed an extensive, inflamed swelling of almost stony hardness, on the left side of the neck, immediately over the clavicle, which, treated with emollient applications, passed rapidly into a copious and deep suppuration of the cellular tissue. Large sloughs of the cellular tissue were discharged with laudable pus, so that after the entire removal of these, the muscles of the neck could be seen as if dissected out. The entire healing of this deep wound was completed about the end of September.

Dr. W. thinks, that the hyd. potass. not only produced a critical discharge in the copious secretion of urine, but that it also, perhaps, occasioned a beneficial revulsion by causing the swelling above mentioned to suppurate. He thinks, also, that the mercury which had been taken, may have contributed to produce this favorable result.—*Oppenheim's Zeitschrift, für die gesammte Medicin*, April, 1843.—*Am. Jour. Med. Sci.*

